# Score regularization for peptide identification

**DOI:** 10.1186/1471-2105-12-S1-S2

**Published:** 2011-02-15

**Authors:** Zengyou He, Hongyu Zhao, Weichuan Yu

**Affiliations:** 1 School of Software, Dalian University of Technology, Dalian, China; 2 Department of Epidemiology and Public Health, Yale University, New Haven, Connecticut 06520, USA; 3 Laboratory for Bioinformatics and Computational Biology, Department of Electronic and Computer Engineering, The Hong Kong University of Science and Technology, Hong Kong, China

## Abstract

**Background:**

Peptide identification from tandem mass spectrometry (MS/MS) data is one of the most important problems in computational proteomics. This technique relies heavily on the accurate assessment of the quality of peptide-spectrum matches (PSMs). However, current MS technology and PSM scoring algorithm are far from perfect, leading to the generation of incorrect peptide-spectrum pairs. Thus, it is critical to develop new post-processing techniques that can distinguish true identifications from false identifications effectively.

**Results:**

In this paper, we present a consistency-based PSM re-ranking method to improve the initial identification results. This method uses one additional assumption that two peptides belonging to the same protein should be correlated to each other. We formulate an optimization problem that embraces two objectives through regularization: the smoothing consistency among scores of correlated peptides and the fitting consistency between new scores and initial scores. This optimization problem can be solved analytically. The experimental study on several real MS/MS data sets shows that this re-ranking method improves the identification performance.

**Conclusions:**

The score regularization method can be used as a general post-processing step for improving peptide identifications. Source codes and data sets are available at: http://bioinformatics.ust.hk/SRPI.rar.

## Background

The identification of peptides by searching tandem mass spectrometry (MS/MS) spectra against a protein database is an essential technology in shotgun proteomics. Current peptide search engines such as Mascot [1] and Sequest [2] work on the principle of “query by spectrum”. They mainly use the spectrum-associated information such as peak location (*m/z*), peak intensity and peak types (e.g., *b*-ion, or *y*-ion) to perform peptide identification. Such spectrum-based database searching methods are far from satisfactory since random peptide-spectrum matches (PSMs) occur frequently in the identification results. These false assignments can be attributed to the poor quality of spectra, post-translational modifications (PTMs) of proteins and other unpredictable factors, making it challenging to distinguish correct identifications from incorrect ones.

To improve the identification performance, one possible solution is to incorporate extra information. For example, mass spectrometry is usually coupled with liquid chromatography (LC), which provides retention time measurement associated with the general biophysical characteristics of a peptide. The idea of using retention time for peptide identification has been discussed recently [3].

We note that peptides are correlated with each other. This observation motivates us to exploit the inter-peptide relationship as an additional source of information. The most straightforward and reliable relationship between two peptides is their coexistence in proteins. Two peptides are said to be “related” or “similar” if they belong to the same protein. We define the similarity between two peptides as the probability of their simultaneous occurrence in the same protein. Intuitively, the identification of one peptide will indicate the existence of its related peptides. Therefore, it is reasonable to extend this intuition to the following hypothesis: *Related peptides should have similar ranking scores*. Such a consistency pattern within related peptides can be utilized to re-order PSMs through the manipulation of ranking scores. In this paper, we formulate the consistency-based PSM re-ranking problem as an optimization problem of balancing the score from initial identification against the scores of related peptides. We attempt to unify two contending goals in one single objective function:

1. Smoothing consistency: The PSMs with similar peptides should have similar scores.

2. Fitting consistency: The initial ranking score provides valuable information. Thus, the new score of each PSM should not deviate too much from its original one.

Here we use a linear combination of these two objectives and introduce a regularization parameter to control their relative importance. This optimization problem has a closed-form solution. We apply the proposed method to several real MS/MS data sets. Experimental results show that our method consistently outperforms the baseline method.

The rest of the paper is organized as follows: Section 2 presents the problem formulation and our method. Section 3 shows the experimental results. Section 4 discusses related methods and Section 5 concludes the paper.

## Methods

In this section, we first introduce the problem and illustrate the underlying assumption using a motivating example. Then, we present a probability-based similarity measure to quantify the inter-peptide affinity. Finally, we provide an optimization formulation under the regularization framework and discuss how to find the optimal solution efficiently.

### Problem statement

Let ℂ = {(*p*_1_, *s*_1_), (*p*_2_, *s*_2_), …, (*p_n_*, *s_n_*)} be a set of PSMs, where *p_i_* is a peptide sequence and *s_i_* is a MS/MS spectrum. The set ℂ is associated with a vector of initial ranking scores *X* = (*x*_1_, *x*_2_, …, *x_n_*)*^T^* provided by a standard peptide search engine (i.e., baseline ranker). There is no special requirement about the input ranking score, i.e., it may be any type of score (e.g., probability score, E-value).

As the baseline ranker tends to be imperfect, the goal of our re-ranking method is to find a new score vector *Y* = (*y*_1_, *y*_2_, …, *y_n_*)*^T^* using the inter-peptide relationship to improve the ranking.

Note that it is possible to have *p_i_* = *p_j_* for *i* ≠ *j* since the same peptide may be identified by multiple spectra.

### A motivating example

Practically, the assumption that “peptides from the same protein should have similar ranking scores” is often violated. This is because different peptide sequences may have different physicochemical properties and fragmentation pattern, which will result in different scores from database search engines, even if they are from the same protein. However, imposing such a consistency constraint is still beneficial for peptide identification. We shall explain our idea using a toy example.

**Figure 1:**
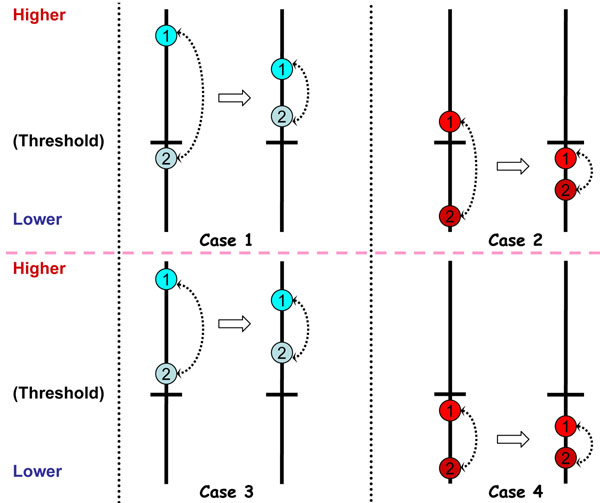
**An illustration of the effect of using consistency constraint in PSM re-ranking.** Here each solid circle represents a hypothetical peptide-spectrum pair, whose position is proportional to the ranking score. The consistency constraint shortens the distance between two related PSMs to increase their mutual affinity, leading to new ranking scores. If we consider the PSMs above the threshold to be correct, the 2nd PSM in case 1 becomes correct and the 1st PSM in case 2 becomes incorrect after the transformation. In case 3 and case 4, the consistency constraint has no effect on the correctness state of PSMs.

Suppose a hypothetical protein consists of two detected peptides. Fig.1 shows four possibilities according to the distribution of ranking scores and a pre-defined threshold value.

• Case 1: One PSM has a very high score and the other PSM has a score below the threshold. The consistency constraint will force them to move towards each other in terms of ranking scores. This may provide the second PSM a higher score above the threshold. This change will improve the identification performance if the second PSM is correct, but will lower the performance otherwise. Fortunately the probability that the second PSM is correct is higher since the probability that its parent protein exists is high (though the first peptide might be identified simply due to chance, it is unlikely that such identification will have a high score).

• Case 2: The score of first PSM is barely above the threshold and that of second PSM is very low. Penalizing their scores according to the consistency assumption will pull down the first PSM (below the threshold). Though the constituent peptide-level match scores of a truly present protein may vary widely, it is unlikely that all these scores are not high. Therefore, the probability that the first PSM is incorrect is high. In other words, there is a good possibility to detect this incorrect identification via consistency-based score adjustment.

• Case 3 and Case 4: The result will not change.

The above example shows that penalizing inconsistent PSMs per protein may help us improve the initial identification performance. This observation motivates us to investigate the possibility of utilizing consistency hypothesis in PSM re-ranking, even when such an assumption does not always hold in real proteomics experiments.

### Graph construction

Each peptide may belong to multiple proteins. We use *U_i_* to denote the set of proteins that contains *p_i_.* Given *n* peptide-spectrum pairs, we construct an *n* × *n* symmetric similarity matrix *W* with its element *w_ij_* measuring the similarity between *p_i_* and *p_j_.* Concretely, *w_ij_* is defined as the probability that these two peptides belong to the same protein:(1)

where *U_i_* ∩ *U_j_* denotes the set of proteins that contains both *p_i_* and *p_j_*, and |·| denotes the number of elements in a set.

Given *W*, we define a diagonal matrix *D* as:(2)

The matrix *D* will be used in the next subsection.

**Figure 2:**
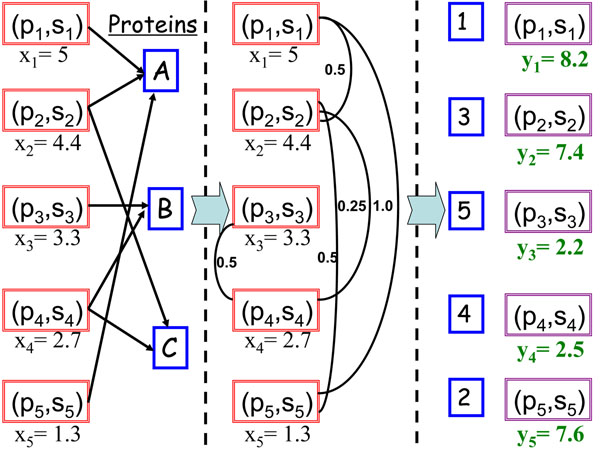
**An illustration of graph construction and PSM re-ranking**. In this example, we have 5 peptide-spectrum pairs and 3 proteins (left). According to the peptide-protein relationship, we generate an affinity graph with 5 edges (middle). We use the proposed method to generate new ranking scores when *λ* is set to 0.6 (right).

In Fig.2, we use a toy example to describe the graph construction procedure. In this example, there are five peptide-spectrum pairs and three proteins {*A*, *B*, *C*}*.* According to the peptide-protein relationship in the left part of Fig.2, we obtain *U_i_* for each peptide *p_i_*:

*U*_1_ = {*A*}, *U*_2_ = {*A*, *C*}, *U*_3_ = {*B*}, *U*_4_ = {*B*, *C*}, *U*_5_ = {*A*}*.*

Then, we construct a similarity graph as shown in the middle part of Fig.2. The corresponding *W* and *D* read:

Here are some characteristics of *W:*

• It is a sparse matrix, which can be loaded using a relatively small storage space.

• The number of non-zero entries in each row varies significantly. This is because one peptide may have many neighbors while another peptide may have only a few neighbors.

• There is no self-loops in the graph since its diagonal entries are zeros *(w_ii_* = 0).

### Regularization framework

Given the vector of initial scores *X* and the similarity matrix *W*, we compute a vector of new scores *Y* for the same set of PSMs by considering two objectives: smoothing consistency among similar peptides and fitting consistency between new scores and initial identification scores.

#### Smoothing consistency

We use the following cost function to quantify the inter-peptide inconsistency:(3)

where *d_ii_* and *d_jj_* are the diagonal elements of matrix *D.*

If related peptides (*w_ij_* > 0) have very inconsistent scores, then the value of *L*(*Y*) will be high.

It is important to mention the following equation [4]:(4)

In the spectral graph theory [5], *I* – *D*^–1/2^*WD*^–1/2^ is called the normalized Laplacian. The appendix gives the detailed proof of equation (4) and corresponding interpretations based on the spectral graph theory.

#### Fitting consistency

We define another cost function to penalize the inconsistency between the initial identification score and the new score:(5)

If peptides have scores that are inconsistent with their original scores, then the value of *F*(*Y*) will be high.

#### Score regularization

We use a linear combination of *L*(*Y*) and *F*(*Y*) to compose the regularized objective function:(6)

where λ ∈ (0*,*1) is a regularization parameter controlling the balance between the smoothing consistency and the fitting consistency.

Thus, the consistency-based PSM re-ranking problem is formulated as finding an optimal Y* such that:(7)

The above optimization problem has been studied in machine learning [6]. It has a closed-form solution. Concretely, we set the derivative of *Q*(*Y*) (with respect to *Y*) equal to zero:(8)

where *S* = *D*^–1/2^*WD*^–1/2^ and *I* is the identity matrix.

After some algebraic derivation, we obtain the closed-form solution (see the appendix for the rigorous proof that the inverse always exists):

Y* = *λ*(*I* – (1 – *λ*)*S*)^–1^*X.*        (9)

For the toy example shown in Fig.2, the closed-form solution gives a new ranking list (see the right part of Fig.2).

### Miscellaneous issues

#### Isolated nodes

To compute the closed-form solution, each *d* in *D* can not be zero. In other words, there should be at least one non-zero entry in each row of *W.* This means that each peptide must have some similar peptides in the graph. For those isolated peptides, we have two choices:

• Exclude these peptides during graph construction, but keep their identification scores during re-ranking.

• Introduce a dummy node as the neighbor of each isolated node. Meanwhile, set the corresponding similarity value to an extremely small positive number (e.g., 1*/*10^8^).

In this paper, we adopt the second strategy for the sake of implementation simplicity.

#### Large-scale implementation

The matrix *S* is usually very sparse and needs a relatively small storage space. However, (*I* – (1 – *λ*)*S*)^–1^ may be very dense and requires a huge storage space. When the computation of (*I* – (1 – *λ*)*S*)^–1^ is infeasible due to space limitation, we use the following iteration [6] to find the solution:

*Y*(*t +* 1) = *λX +* (1 – *λ*)*SY*(*t*).        (10)

It has been proved that the iteration process will converge to the closed-form solution Y*[6]. Since *S* is sparse, this method requires less storage space than computing the closed-form solution directly. Intuitively, the iteration can be understood as an information diffusion process on the graph. In each round, every node updates its score by linearly combining its own score and the scores of its neighbors.

#### Protein inference

Peptide identification is only one intermediate step of protein identification. Though the PSM re-ranking strategy is able to effectively improve peptide identifications, one may wonder if it really helps in protein identification. Indeed, the fact that better peptide re-ranking results will lead to better protein inference have been experimentally verified for several times (e.g., [7]). Therefore, we will focus on the identification performance comparison at the peptide level in this paper.

## Results

We apply our method to four real data sets (named DS1-DS4) in Table 1. We use X!Tandem [8][version 2007.07.01.2] as the baseline ranker to search against a composite target-decoy database. In the composite database, we use proteins from the Swiss-Prot database (release 56.6) as target entries and shuffle these target protein sequences to generate decoy entries. Each decoy protein sequence is a random permutation of residues in the corresponding target protein. Here we use **T** and ℝ to denote the set of target proteins and the set of decoy proteins, respectively.

**Table 1:** Description of MS/MS data sets used in the experiment.

*Name*	*Source*
DS1	ISB standard protein mixture [17]
DS2	ABRF sPRG2006 protein mixture
DS3	Human serum protein mixture [18]
DS4	ABRF sPRG2009 protein mixture

Their URLs and file names are provided in the appendix.

We use the following parameters for peptide identification: mono-isotopic masses, mass tolerance of 2Da for precursor, mass tolerance of 1Da for fragment ion, fixed modification on Cys and one missed cleavage site. We only consider *b* and *y* fragment ions in PSM scoring. We use the negative logarithm of E-value of each PSM provided by X!Tandem as the initial ranking score. The criterion for filtering PSMs is E-value ≤ 0*.*1. Our method generates a set of new scores that better distinguishes correct identifications from incorrect ones. Through the experiment, the regularization parameter *λ* is fixed to 0.5 unless it is explicitly specified. In performance evaluation, a peptide-spectrum pair (*p_i_,x_i_*) is labeled as a false positive if *p_i_* belongs to a decoy protein; otherwise, it is a true positive. Given a vector of ranking scores *X* = {*x*_1_, *x*_2_,…, *x_n_*} and a score threshold *δ*, the true positive rate (TPR) is defined as the number of true positives above the threshold divided by the total number of true positives:

*TRP*(*X*, *δ*) = |{*p_i_* ∈ **T**|*x_i_* ≥ *δ*}|/|{*p_i_* ∈ **T**}|.        (11)

Similarly, the false positive rate (FPR) is defined as:

*FPR*(*X*, *δ*) = |{*p_i_* ∈ ℝ|*x_i_* ≥ *δ*}|/|{*p_i_* ∈ ℝ}|.        (12)

**Figure 3:**
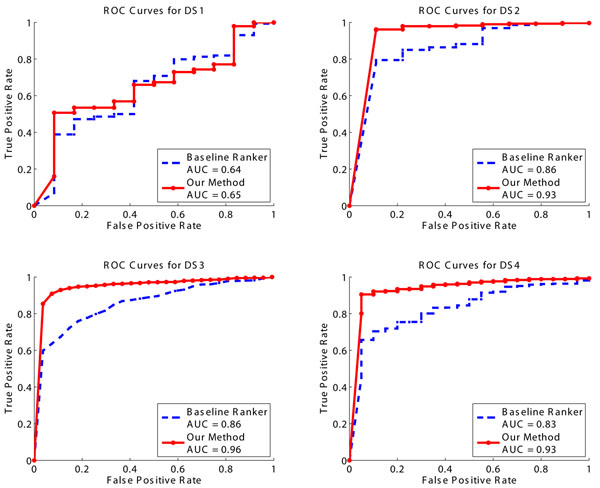
**Identification performance of baseline ranker and our method.** Here we use X!Tandem as the baseline ranker to rank PSMs according to their E-values. Our method outputs an optimal ranking that balances the score consistency among similar peptides and the score consistency between initial identification and updated identification.

We plot the receiver operating characteristic (ROC) curves of the baseline method and our method in Fig.3. We also use the area under ROC curve (AUC) as a single numeric indicator of overall performance. Fig.3 shows that:

1. Our consistency-based re-ranking method provides consistent and substantial performance improvement on the data set DS2, DS3 and DS4. Note that our method does not require any prior knowledge or training data.

2. Though there is only a marginal improvement of the overall performance on DS1 (AUC=0.64 vs. AUC=0.65), we note that our method achieves significantly higher true positive rate than the baseline method when the false positive rate is around 10%. It is a nice property since the false positive rate is usually set to a relatively small value in practice.

**Figure 4:**
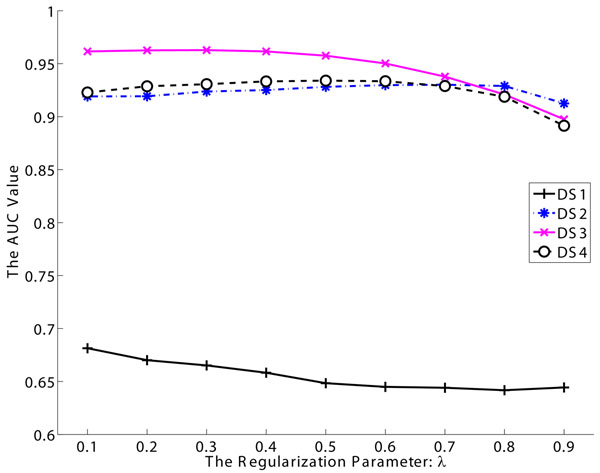
**Effect of regularization parameter on the identification performance in terms of AUC**. Here *λ* ranges from 0.1 to 0.9.

To test the sensitivity of our algorithm to the regularization parameter, we vary *λ* from 0.1 to 0.9 and plot the AUC values in Fig.4. It shows that the identification result is robust with respect to *λ*. The increase of *λ* (when *λ* > 0*.*6) will lead to the decrease of AUC since the regularized score will be identical to the initial score when *λ* = 1. Though we cannot determine the optimal *λ* automatically, we suggest to set *λ* = 0*.*5 as a rule of thumb in practice since this setting exhibits good performance on average.

**Figure 5:**
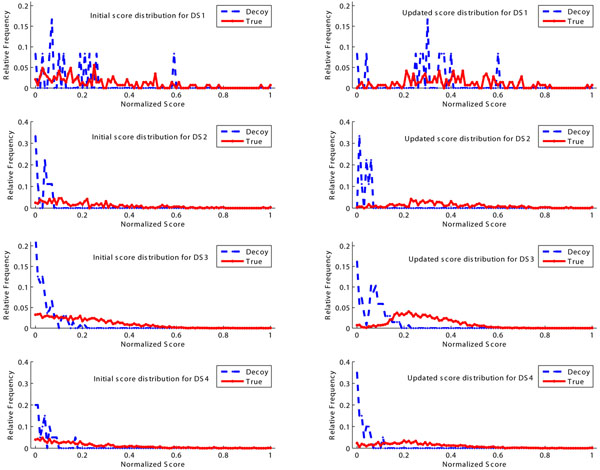
**The score distribution before and after re-ranking**. Left: The score distribution of true identifications and decoy identifications before re-ranking. Right: The score distribution of true identifications and decoy identifications after re-ranking. Both the initial score and updated score are normalized into the interval [0,1] with a min-max normalization procedure.

We also plot the initial score distribution and the updated score distribution in Fig.5. Here we use the min-max normalization to transform both the initial identification score and the new re-ranked score into the interval [0,1]. It reveals that the consistency constraint will shrink scores in each group (true and decoy) towards their mean value. Although the consistency-based re-ranking method cannot completely separate true identifications from decoys, it does reduce the score overlap on DS2, DS3 and DS4. Note that the consistency-based re-ranking procedure is less effective on DS1 since there is a serious score overlap. Even in this case, we find that the separation between true and decoy identifications is improved at lower score region.

## Discussion

Here we briefly review previous works related to the ideas discussed in this paper.

### PSM re-ranking

Many PSM scoring algorithms have been developed to facilitate accurate peptide identification. Mascot [1], Sequest [2] and X!Tandem [8] are the mostly used PSM scoring algorithms. These algorithms use information in each single MS/MS spectrum to perform peptide inference. As discussed in the introduction, they suffer from the problem of generating incorrect PSMs due to various reasons. An effective post-processing strategy is to re-order PSMs so as to reduce the overlap between correct identifications and incorrect identifications.

Machine learning techniques are widely used to build re-ranking models [9-14]. These methods require high-quality MS/MS spectra as training data to generate an accurate classification or regression model.However, it is very difficult to obtain a discriminative model that is universally applicable to different platforms and experimental conditions.

One may argue that some semi-supervised learning methods such as Percolator [10] do not require any training data. This is not true since they still need to build a predictive model, in which the training set is constructed automatically on the fly: The PSMs derived from searching a decoy database are used as negative examples and the high-scoring PSMs derived from searching the target database are used as positive examples. Eliminating the need of constructing a training set manually cannot be interpreted as being free of training data.

The proposed method is similar to those learning-based re-ranking approaches in the sense that it borrows information from different spectra. The novelty that distinguishes our method from previous ones is that we explicitly exploit the rank dependence between/among peptides from the same protein.

Overall, the consistency-based re-ranking model offers several advantages:

1. It does not need MS/MS spectra as training data. This flexibility makes the algorithm applicable to MS/MS data generated from different platforms and experimental conditions.

2. It utilizes the inter-peptide relationship during the re-ranking process. Such information is readily available in the protein database. Furthermore, this peptide-peptide connection encoded in protein sequence is very stable and noise-free.

3. The optimization problem in this paper has a closed-form solution, which enables us to obtain the optimal re-ranking list easily.

### Discrete regularization

The idea of regularization has been widely studied in the literature. In particular, similar optimization formulations have been used in semi-supervised learning [6] and information retrieval [15]. To the best of our knowledge, there has been no previous work that applies this idea to peptide identification and PSM re-ranking.

Since we use the regularization technique in a different problem setting, some subtle differences among different methods exist. For instance, the methods in machine learning [6] and document retrieval [15] usually generate equal number of neighbors (i.e., the number of non-zero entries in the row of *W*) for each node. While the number of neighbors of different peptides in our similarity matrix may vary significantly.

### Peptide dependency

The idea of incorporating dependencies of peptides from the same protein has been used in ProteinProphet [16]. Here we highlight that there are at least three key differences between our formulation and ProteinProphet.

1. Our objective is to re-rank PSMs while ProteinProphet aims at protein inference.

2. Our method can lower the score of a high scoring PSM in the presence of low scoring matches from the same protein. This will improve the identifications of some one-hit wonders. Otherwise, they may be overwhelmed. In contrast, the adjustment mechanism in ProteinProphet favors peptides having many neighbors.

3. We can find the optimal solution while ProteinProphet doesn’t has such a property.

## Conclusions

This paper introduced a consistency-based PSM re-ranking method: Given an initial set of identification scores and the inter-peptide similarity matrix (graph), the new method finds a set of new scores by minimizing the score inconsistency among similar peptides and the score inconsistency between updated identification and initial identification. Since the new method only requires the initial identifications as input, we can apply it to initial rankings from any peptide search engines. Thus, this consistency-based score regularization can be used as a general post-processing step in peptide identifications.

The affinity measure in this paper only considers inter-peptide relationship and ignores other sources of information contained in the peptide-spectrum pairs. For instance, many valuable features such as peak offset and sequence composition can help us define more comprehensive similarity metrics. Such extensions will generate an enhanced affinity graph since two peptide-spectrum pairs may become similar even when their peptides do not belong to the same protein. We will study whether such an extended graph model can further improve the identification performance in the future work.

Our model is based on the hypothesis that “similar peptides should have similar ranking scores”. This hypothesis can have different interpretations, making it possible to formulate different optimization problems. For example, we can use the relative rank instead of the ranking score in the objective function. The investigation of alternative optimization formulations is another interesting topic.

## Competing interests

The authors declare that they have no competing interests.

## Authors contributions

ZH performed the implementations and drafted the manuscript. HZ and WY conceived the study and finalized the manuscript. All authors read and approved the final manuscript.

## Appendix

### Rationale Behind Normalized Laplacian

In this section, we present the rationale of using normalized Laplacian in the cost function of smoothing inconsistency.

To penalize the smoothing inconsistency, one straightforward method is to use the weighted squared difference among scores of similar peptides:(13)

The spectral graph theory [4] shows that the following equation holds:(14)

In the spectral graph theory, *D* – *W* is the unnormalized Laplacian [5]. Recall that different peptides may have different number of neighbors, the cost function based on unnormalized Laplacian will place more penalties on those peptides with more neighbors. To address this issue, we use the *normalized* Laplacian to replace the *unnormalized* Laplacian in *L*(*Y*).

### Detailed Data Description

In Table 2, we list the URLs and file names of the MS/MS data used in the experiment.

**Table 2:** URLs and names of MS/MS data files.

*Name*	*URL*	*File Name*
DS1	http://regis-web.systemsbiology.net/PublicDatasets/	The 2nd replicate of mixture 2 (QSTAR)
DS2	https://proteomecommons.org/dataset.jsp?i=65964	Lane/060121Yrasprg051025-ct5.RAW
DS3	http://www.peptideatlas.org/repository/	PAe000330/1021505_LTQ10401_1_2.mzXML
DS4	https://proteomecommons.org/dataset.jsp?i=66047	sh_072808p_E_coli_ABRF_red.mzXML

### Some Proofs

Theorem 1 and Theorem 2 imply the positive semi-definiteness of unnormalized Laplacian and normalized Laplacian, respectively.

**Theorem 1.***For every vector Y* , *we have*(15)

*Proof.* Here we repeat the proof of [4] for completeness.

**Theorem 2.***For every vector Y* , *we have*(16)

Proof.

To compute the closed-form solution, the matrix *(I* – *αS)* must be invertible, where *α* = 1 – *λ*. Here we provide the detailed proof for the sake of completeness since it is omitted by [6].

Before we proceed to prove the existence of the inverse, we first show that the following two lemmas are correct.

**Lemma 1.***Both* (*I* – *S*) *and* (*I + S*) *are positive semi-definite*, *where S* = *D*^–1/2^*WD*^–1/2^*.*

*Proof.* According to Theorem 2, *(I* – *S)* is positive semi-definite since  is always non-negative.

Similarly, we can show that. . Thus, *(I + S)* is also positive semi-definite.

**Lemma 2.***The eigenvalues of matrix S* = *D*^–1/2^*WD*^–1/2^*fall into [-1,1].*

*Proof.* According to Lemma 1, (*I* – *S*) is positive semi-definite. Meanwhile, *(I* – *S)* is symmetric. Thus, all the eigenvalues of (*I* – *S*) are nonnegative. Let *e* be an eigenvalue of *S* and the associated eigenvector is *V*, i.e., *SV* = *eV.* Then,

(*I* – *S*)*V* = *V* – *SV* = *V* – *eV=*(1 – *e*)*V.*

Therefore, (1 – *e*) is the eigenvalue of (*I* – *S*) and *V* is its corresponding eigenvector. Thus, 1 – *e* ≥ 0, i.e.,all the eigenvalues of *S* are not larger than 1.

Similarly, we can prove that each eigenvalue of *S* is at least -1.

**Theorem 3.** (*I* – *αS*) *is invertible*, *where S* = *D*^–1/2^*WD*^–1/2^*and α* ∈ (0, 1)*.*

*Proof.* A matrix is invertible if and only if all its eigenvalues are non-zero. Here we show that each eigenvalue of (*I* – *αS*) is non-zero.

Let *e* be an eigenvalue of *S* and the associated eigenvector is *V.* Then,

(*I* – *αS*)*V* = *V* – *αSV* = *V* – *αeV=* (1 – *αe)V.*

Obviously, 1 – *αe* is the eigenvalue of (*I* – *αS*) and *V* is the corresponding eigenvector. According to Lemma 2, we know that –1 ≤ *e* ≤ 1. Thus, 1 – *αe* > 0 holds since *α* ∈ (0*,*1).
